# Intention for international assignment among workers in Ghana: Modelling the role of motivators, demotivators and cultural disposition

**DOI:** 10.1371/journal.pone.0284615

**Published:** 2023-05-04

**Authors:** Moses Segbenya, Nana Yaw Oppong

**Affiliations:** 1 Department of Business Studies, College of Distance Education, University of Cape Coast, Cape Coast, Ghana; 2 Department of Human Resource Management, School of Business, University of Cape Coast, Cape Coast, Ghana; Babes-Bolyai University, Cluj-Napoca, ROMANIA

## Abstract

This study examined intention for international assignment among workers in Ghana by modelling the role of motivators, demotivators and cultural disposition on such intentions. The cross-sectional survey design was used to sample 723 workers drawn from Northern Ghana. Data were collected with a self-administered questionnaire. The Partial Least Square-Structural Equation Modelling was used to analyse the data collected. The study found from individual workers’ and developing economy perspectives that cultural disposition influences motivation for accepting international assignments and expatriates’ intention to accept international assignments. Motivation and demotivation among workers were also found to have had a statistically significant relationship between expatriate intention and significantly mediated the relationship between cultural disposition and expatriate intention to participate in international assignments. Cultural disposition was, however, found to have a non-significance relationship with expatriates’ intention to accept an international assignment. It is therefore recommended that human resource managers should make international assignments attractive for workers and expose workers to cross-cultural training through job rotations, working in teams and experiential training. It is expected that such opportunities prepare individuals for an international assignment.

## Introduction

The competitive business environment continues to compel organisations to spread their activities abroad for growth. Multinational and transnational organisations have resulted in deploying workers/managers to international locations to push their organisational goals and objectives [1]. Assignee managers or staff, either with or without families, take up such responsibilities and positions in another country for a period of six months to five years [2, 3]. Thus, international assignment in this context relates to dispatching employee/s by their organisation from their home country to take up an oversea task temporarily to work at overseas offices or companies. International assignments, thus, help organisations to promote global integration and competencies and build a global and mature workforce [4, 5].

Meanwhile, the ability of organisations to achieve targets associated with international assignments largely depends on the success of assignees on the international assignment [6]. Several factors play influential roles in determining the success of expatriates or staff on international assignments [6, 7]. Success in international assignments starts with the intention and motivation to take up an international assignment [8]. Studies have found that international positions and appointments not based on the motivation of assignees are often not successful [7–9]. Thus, human resource managers of multinational organisations need to pay attention to motivation among staff to take up an international assignment [2, 10]. Factors that can motivate staff to take up international assignments can include remuneration, support for spouses and children and good organisational policies on returning to the home country after an international assignment [7, 11, 12]. It stands to reason that in the absence of motivators, workers are likely going to be disinterested or demotivated to take up an international assignment.

Motivation or demotivation is not enough to explain the success of expatriates. For this reason, cultural disposition was found to have played a critical role in the success of international assignees [6]. Cultural disposition relates to the exposure of the assignee to other cultures, especially the culture of the host country of the international assignment [6]. The ability of expatriates to express themselves in the host country’s language and other aspects of its culture has the potential to ensure the success of expatriates [6]. Thus, cross-cultural training could play a critical role in enhancing the chances of an expatriate’s performance in an international assignment [8].

Ghana hosts branches or headquarters of several multinational and transnational organisations. Therefore, the human resource of these organisations can be described as either host country, home country, or third-country national. The filling of vacancies in overseas subsidiaries will continue to be an important issue for international human resource managers for the growth of their organisations [6, 13]. While some workers are already exposed to international experience, others have yet to have such opportunities [3, 14]. Those who have had expatriate or international experience by working on international assignments could have also learnt their lessons as to whether to take up a future international assignment of their organisations (expatriation or im-patriations) or other organisations in the form of self-initiated oversea assignment.

In this regard, workers’ intention towards an international assignment is key for international human resource managers to recruit for international assignments [14, 15]. Workers’ intentions could be influenced by their cultural disposition and motivation to accept international assignments [11]. Alternatively, certain demotivators and failure in an earlier international assignment could explain the lack of motivation for an international assignment [8, 13, 16]. International human resource managers benefit from the knowledge of motivators or demotivators among workers who have international working exposure and those who do not.

Meanwhile, earlier studies by [17–23] have focused on cultural adjustment and expatriate performance, job satisfaction, expatriate’s spouse adjustment; and [22, 24] on job insecurity, expatriate’s benefits. Though [24–27] delved into an intention to accept an international assignment, these studies were conducted from the organisational approach. Thus, individual and contextual (Ghanaian) perspectives were either not addressed or not adequately addressed. This is because individuals’ appreciation of motivating and demotivating factors as well as their disposition towards various cultures could differently influence their intention to accept international/expatriate assignments. It is also possible that individuals’ socio-demographics (either married or single, number of foreign languages individuals can speak) could influence workers’ decision to accept expatriate assignments. The individual perspective is deemed very important for this study because it could not just influence the intention to accept an international assignment but also ensure higher employee performance in an international assignment.

The novelty and motivation for this study are not limited to the individual perspective but also to bring to the fore how cultural disposition, motivating, and demotivating factors from a developing economy perspective are influencing acceptance of international assignments. For this reason, this study sought to contribute to the literature on international assignment and international human resource management by examining the intention for international assignment among workers in Ghana. This study specifically modelled the role of motivators, demotivators, and cultural disposition in predicting intention to accept an international assignment from individual and developing economy perspectives. Thus, the role of factors that could serve as motivators and demotivators, as well as the cultural disposition of workers, were examined from workers’ perspectives in terms of how these factors could influence their intention and performance. The next sections of the paper focus on the theoretical review, conceptual model and hypotheses development. Other issues addressed were the methodology, results and analysis, discussion of the results, implications, conclusions and recommendations.

## Literature review

### Theoretical review

This study was guided by the Hofstede’s Cultural Model. The cultural model propounded by Geert Hofstede in 1980 was based on five pillars-*power distance*, *individualism*, *masculinity; uncertainty avoidance; and long-term orientation* [28, 29]. Each of these five pillars has two dimensions-high or low, suggesting that the level of individual workers’ inclination towards these pillars could influence the intention to accept international assignments. The first pillar, which is ***power distance*,** relates to power distribution between subordinates and superiors or society. The subordinates and superiors are the same in low power distance society. However, in a high-power distance society like the study context (Ghana), power is unevenly distributed or centralised among a few people. Hence superiors are different from subordinates because the superiors have power, and subordinates need to accept and respect it [29].

*The* second pillar of Hofstede’s Cultural Model—*Individualism/Collectivism*, explains how individuals relate to a group. A society where individuals tend to focus on their own interests and that of the immediate family relates to an individualised culture [29]. However, where individuals respect and seek the good and respect the group that they belong, it is a collectivist society. The third dimension, *masculinity or femininity*, deals with what motivates people. In masculine society, people strive to be the best; they portray values such as competitiveness and performance in that masculine have sympathy for the successful achievers, However, in feminine culture, people have a concern with relationships and quality of life [29].

The fourth dimension-*Uncertainty Avoidance-* relates to the level to which individuals in society welcome uncertainty [28]. In high uncertainty avoidance society, individuals are afraid to take a risk for fear of failure, unlike in a low uncertainty avoidance society where individuals are ready to deal with any situation regardless of the outcome. The last dimension of the theory, termed as *short/long-term orientation*, is also explained the extent to which society shows a future-oriented perspective versus a short-term point of view. Thus, short-term profit concentration characterised society with short-term orientation whiles people of long-term orientation emphasise future growth. The Hofstede’s cultural model with the five dimensions revealed that the Ghanaian society that serves as the study context is largely inclined towards a high-power distance culture, collectivism culture, feminine culture, high uncertainty avoidance culture, and short-term orientation culture [28, 29].

### Conceptual model and hypotheses development

The discussion in this section will focus on the key variables of the study, which are cultural disposition, motivation, demotivation, and intention to accept an international assignment among workers. The review for each subsection will be associated with the specific hypothesis related to it.

#### Cultural disposition and intention for international assignment

Culture relates to a way of life and is described as “the collective programming of the mind which differentiates one group from the other” [28]. Culture could be classified into material, tangible and non-material or intangible cultures. Key components of culture are the structure of society, values, religion, education, personal communication, and physical environment. Generally, culture has the characteristics of being learned. Language as an essential component, sometimes abstract, a product of behaviour and shared by individuals. Other characteristics also include culture being shared by members of society; it is pervasive and variable. In terms of layers, culture has three basic layers -assumption/beliefs, norms, and values and lastly, behavioural or explicit layers. Thus, culture could be a national culture, organisational culture, corporate culture or professional culture. Workers will easily and gladly accept to work in jurisdictions with similar cultural settings [3, 29]. Some workers see different cultural settings as problematic places requiring so much time and effort to acclimate if they accept an international assignment in such locations [29]. This means that an individual’s cultural disposition plays a role in accepting or rejecting an international assignment [5, 30]. It also means that cultural disposition could be a motivator for contemplating accepting an international assignment [30]. It is for these reasons that this study hypothesised that:

1. *H*_*1*_: *Cultural disposition has a statistically significant effect on the intention to participate in an international assignment*.
2. *H*_*1*_: *Cultural disposition has a statistically significant effect on motivation to accept an international assignment*.

#### Demotivators and intention for international assignment

Demotivators to accepting international assignments are factors that make both companies and employees disinterested in expatriation. For this reason, [30] identified factors including adaptability/cross-culture adjustment, spouse and family concerns, and compensation/salary-related issues that are not managed well. [7, 29] identified five factors that could serve as demotivators and lead to expatriate failure. These are *job-related factors comprising r*ole conflict, role novelty, role clarity and role direction, *organisational factors* including organisational culture novelty, social support from co-workers and superiors, and logistical support. Other categorisations were *positional factors*—hierarchical level, functional area and assignment vector; *non-work factors*-culture novelty and spouse/family adjustment; and *individual factors* including self-efficacy, relational and perceptual skills, previous international assignments, and language fluency [27]. If these factors are not framed well in an organisational policy and managed well, employees will not be motivated to take up an international assignment. To further assess how demotivators influence acceptance of an international assignment among workers in a developing economy perspective, this study hypothesised that:

3. *H*_*1*_: *Demotivation among workers toward international assignments has a statistically significant effect on the intention to participate in an international assignment*.

#### Motivation and intention for international assignment

Several studies have found that there are many factors that motivate workers to accept international assignments [24, 29, 31]. These motivators are tax equalisation, temporary living allowances, language and cross-cultural training, overseas healthcare plan, and host-country housing assistance [23, 31]. Other motivating factors include career and repatriation planning, home leave allowances, rest and relaxation leave, spouse job assistance and child education allowance [13, 24]. The Global Mobility Challenge Survey by Ernst and Young revealed the top five incentives that motivate workers to accept international acceptance. These were repatriation assistance, round-trip airfare to return home for family visits, a paid trip to visit the country before agreeing to move there, paid language training, and immigration assistance for a spouse to obtain employment. Thus, managers must be very concerned about using these factors to motivate intention to accept an international assignment. However, how these motivators influence workers in developing economies to accept an international assignment is not clear Meanwhile, motivation to accept an international assignment could also play a mediating role between cultural disposition and intention to accept an international assignment. This means that apart from motivation influencing intention directly, it could also play an indirect role between cultural disposition and intention to accept an international assignment. It is for this reason that this study hypothesis that:

4. *H*_*1*_: *Motivation to accept international assignments among workers has a statistically significant relationship with the intention to participate in an international assignment*.
5. *H*_*1*_: *Motivation to accept international assignments among workers statistically significantly mediate the relationship between cultural disposition and intention to* to participate in an international assignment.

**Fig 1:**
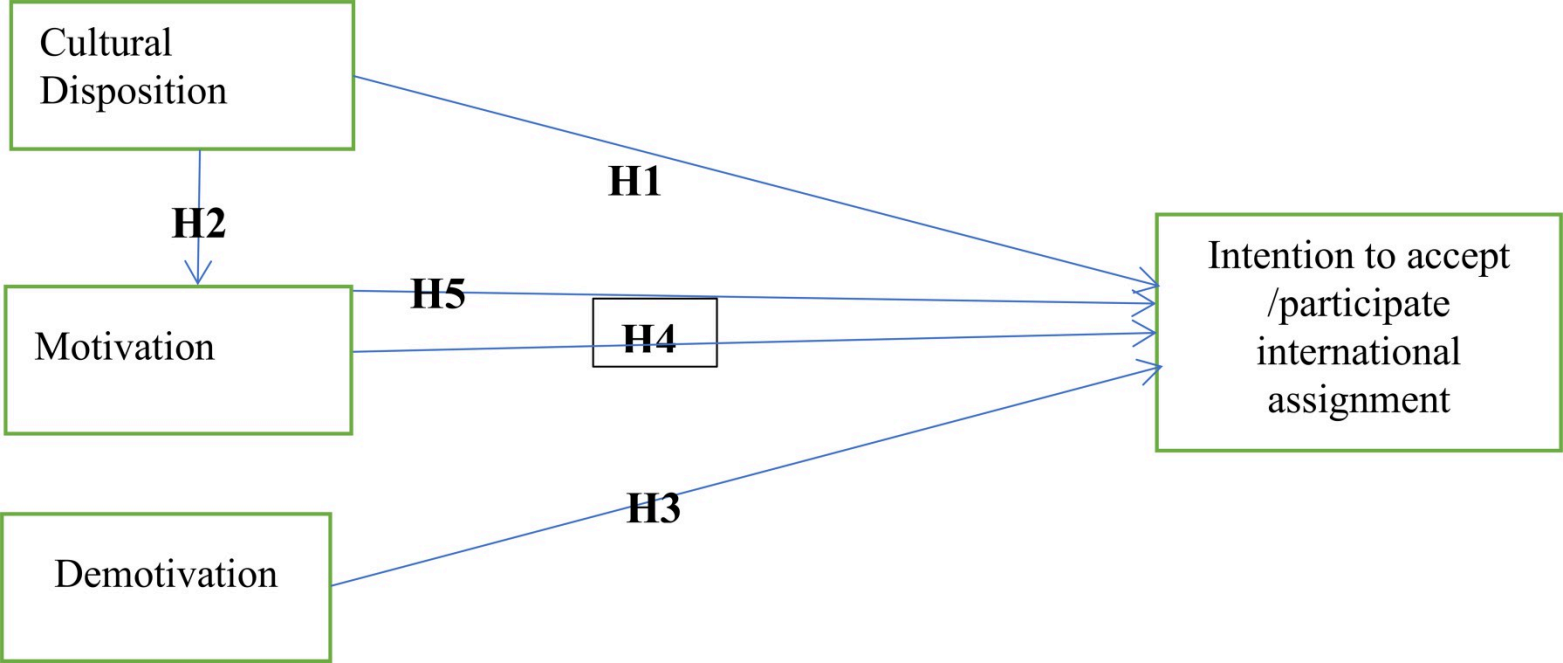
Conceptual framework of the study. Source: Field survey (2021).

Based on the review, a conceptual framework was designed to guide the study, as shown in Fig 1.

### Methodology

The study used a cross-sectional survey design [32]. A sample of 723 workers was drawn from workers in Ghana. Multi-stage sampling techniques [33] including stratified and simple random techniques, were deployed to sample the respondents to the research instrument. These were simple random sampling techniques and stratified sampling techniques. The study adopted the lottery method of the simple random sampling technique. The study employed a multi-stage sampling procedure. The first sampling technique was the simple random sampling technique, and the lottery type of simple random was adopted [34]. The second sampling technique adopted by this study was the proportional stratified sampling technique. Grouping of subjects in a population into the same characteristics (strata) is related to a stratified sample [35]. Another probability sampling technique (cluster or simple random sampling) is deployed for each subgrouping or stratum [34]. The stratified sampling technique was employed because the characteristics of the population were diverse, and there was a need to ensure that every characteristic was adequately represented.

Data were collected, using a self-administered instrument measured on a four Likert point scales where 1 = strongly disagreement, 2 = disagreement, 3 = agreement and 4 = strongly agreement. The questionnaire had two sections. Section one dealt with the biodata of respondents, and section two focused on the five hypotheses guiding the study. The questionnaire obtained a reliability value above the 0.70 thresholds which confirmed that the instrument was good to be used for the data collection. Cronbach alpha values obtained for cultural disposition was .703, motivation was .793, Demotivators was .853, and lastly, intention for international assignment was .868. Data were collected from July 2021 to November 2021. Ethical considerations such as respondents’ anonymity, freedom to withdraw, confidentiality, freedom to participate, and informed consent were observed [36]. Written consent was obtained from respondents prior to data collection. A consent statement was indicated on the questionnaire and participants were to indicate their agreement or disagreement to continue or otherwise with the study.The Partial Least Square Structural Equation Modelling (PLS-SEM) was used to analyse the hypotheses of the study. The PLS-SEM is a method of structural equation modelling which allows estimating complex cause-effect relationship models with latent variables. The PLS-SEM technique is widely applied in business and social sciences, and its ability to model composites and factors makes it a formidable statistical tool for new technology research. The partial least squares structural equation modelling (PLS-SEM) has been said to be the most effective analytical approach for non-experimental research for the last 20 years [37]. Some goodness-of-fit tests that make the PLS-SEM analytical tool very robust are the coefficient of determination (R^2^), effect size (F^2^), and the importance and performance map analysis (IPMA), among others. All these qualities were utilised to make the results of this study very robust.

## Results

### Socio-demographic characteristics of respondents

The first part of the results presented in this section is the s*ocio-demographic characteristics of respondents*. The s*ocio-demographic characteristics* considered in this study, as presented in Table 1, were age, gender, country of birth, sector of employment, international working experience and highest academic qualification. The rest of the s*ocio-demographic characteristics* were marital status, international and local languages spoken, and preferred continent for international job/assignment. The results in Table 1 on respondents’ s*ocio-demographic characteristics* revealed that the majority of the respondents were 30–39 years (48.5%), were male workers (62.0%), Ghanaians by birth (99.3%), public sector workers (82.4%), and never had an international working experience (82.3%). Additionally, the majority of the respondents had tertiary education (88.8%), were married with Kids (54.8%), were fluent in the English language (93.5%), could speak the Dagbani (32%) and Twi local (30.8%) languages, and preferred Europe (48.1%)for their international assignment or job. Though the demographic characteristics are not directly related to the study hypotheses, readers need to understand the characteristics of respondents prior to the presentation of the main findings of the study in the next section.

**Table 1:** Socio-demographic characteristics of respondents.

**1. Age**	**Frequency**	**Percent**
20–29	163	22.5
30–39	351	48.5
40–49	174	24.1
50–59	35	4.8
Total	723	100.0
**2.Gender**		
Male	448	62.0
Female	275	38.0
Total	723	100.0
**3.Country of birth**		
Ghana	718	99.3
Others	5	.7
Total	723	100.0
**4. Employment Sector**		
Public sector	596	82.4
Private sector	90	12.4
Self–employed	37	5.1
Total	723	100.0
**5. International Experience**		
Currently in Ghana on an international assignment	62	8.6
Have ever worked on an international assignment	66	9.1
Never worked on an international assignment	595	82.3
Total	723	100.0
**6. Highest Academic Qualification**		
Primary Education	39	5.4
Secondary Education	42	5.8
Tertiary Education	642	88.8
Total	723	100.0
**7. Marital Status**		
Married without kids	132	18.3
Married with kids	396	54.8
Single	180	24.9
Separated	9	1.2
Widowed	6	.8
Total	723	100.0
**8. International Languages Spoken**		
English	676	93.5
French	19	2.6
German	4	.6
Arabic	22	3.0
Chinese	2	.3
Total	723	100.0
**9. Local Languages Spoken**		
Twi	223	30.8
Ewe	21	2.9
Ga	28	3.9
Dagaare	95	13.1
Dagbani	235	32.5
Gonja	48	6.6
Kasem	46	6.4
Others	27	3.7
Total	723	100.0
**10. Preferred Continent For International Assignment**		
Africa	143	19.8
Europe	348	48.1
South America	83	11.5
North America	83	11.5
Asia	49	6.8
Others	17	2.4
Total	723	100.0

Source: Field survey (2021).

### Model measurement

**Fig 2:**
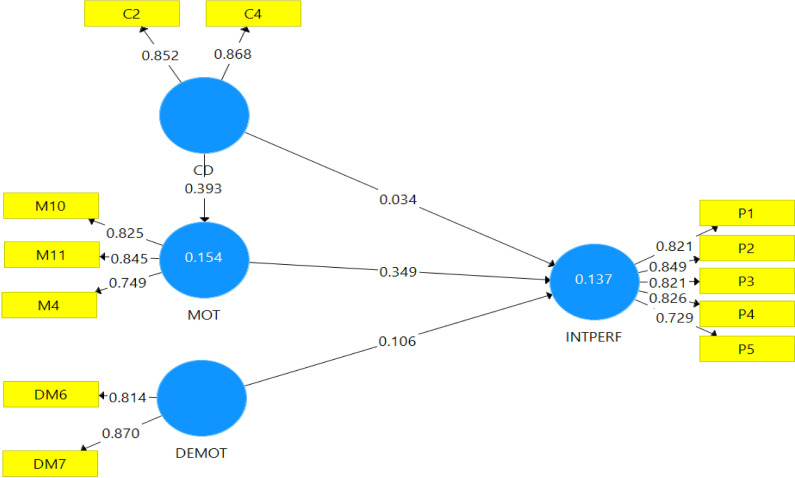
PLS algorithm for confirmatory factor analysis. Source: Field survey (2021).

The estimation of the internal consistency measure of the model was initially carried out with the use of the PLS algorithm for confirmatory factor analysis. The individual items forming each construct or variable in the study were used for the measurement, as seen in the reflective model presented in Fig 2. Based on a minimum threshold of 0.70 for every item to be retained by [38], all items measuring below the minimum 0.70 thresholds were deleted to obtain the results presented in Fig 2. Thus, in Fig 2, the algorithm CFA obtained values above the 0.70 minimum threshold, suggesting that the model achieved internal consistency for the confirmatory factor analysis.

### The measure of internal consistency

The PLS path model’s internal consistency measures were further measured with four indices: rho A, Composite Reliability, Cronbach’s Alpha and Average Variance Extracted (AVE) [38]. Results reported in Table 2 show that Average Variance Extracted (AVE) values obtained for all the constructs ranged from 0.526 to 0.739, and Composite Reliability values were also between 0.810 and 0.905. The last two indices also recorded values ranging between 0.703 and 0.872 for rho -A and 0.701–0.869 for Cronbach’s Alpha. All constructs attained recommended a minimum threshold of 0.70 for Composite Reliability, Cronbach’s Alpha, and rho A [38]. Additionally, [39] recommended a minimum threshold of 0.50 for Average Variance Extracted (AVE) was also attained. Thus, both reliability and validity were attained for all four constructs in the study.

**Table 2:** Construct reliability and validity.

	Cronbach’s Alpha	rho_A	Composite Reliability	Average Variance Extracted (AVE)
CD	0.702	0.703	0.850	0.739
DEMOT	0.701	0.734	0.810	0.592
INTPERF	0.869	0.872	0.905	0.656
MOT	0.774	0.777	0.847	0.526

Source: Field survey (2021).

### Discriminant validity

Heterotrait-Monotrait Ratio (HTMT) was used to ascertain the exclusivity of each construct in the model, as recommended by [40]. The results, as presented in Table 3, show that the diagonal loadings between variables of the study were below 0.85 thresholds and for the same variable was zero [40]. Thus, discriminant validity was achieved for the PLS path model.

**Table 3:** Heterotrait-Monotrait Ratio (HTMT).

	CD	DEMOT	INTPERF	MOT
CD	0			
DEMOT	0.170	0		
INTPERF	0.216	0.087	0	
MOT	0.554	0.133	0.482	0

Source: Field survey (2021).

### Multicollinearity

The inner VIF (variance inflated factors) was used to check the existence of multicollinearity since its existence can fluctuate or affect the results. The recommendations of [38] of VIF values below 3.3 as an indication of the absence of multicollinearity were used, and the results is presented in Table 4. The results revealed that all values of VIF were indeed below 3.3 thresholds, suggesting that there were no multicollinearity issues. Deatialed outer VIF values can be seen from S2 Appendix.

**Table 4:** Path analysis and hypotheses testing.

	R Square					R Square Adjusted			
INTPERF	0.137					0.133			
MOT	0.154					0.153			
						**Confidence Intervals**			**^**Inner VIF**^**
** **	**Beta**	**Sample Mean**	**Standard Deviation**	**T Statistics**	**P Values**	**97.5%**	**2.5%**	**f** ^**2**^	
1. CD -> INTPERF	0.034	0.036	0.036	0.930	**0.353**	0.107	0.031	**0.001**	1.190
2. CD -> MOT	0.393	0.393	0.038	10.235	**0.000** **	0.468	0.317	**0.183**	1.000
3. DEMOT -> INTPERF	0.106	0.113	0.041	2.604	**0.009** **	0.194	0.028	**0.013**	1.010
4. MOT -> INTPERF	0.137	0.138	0.020	6.902	**0.000** **	0.419	0.280	0.119	1.189
**Specific Indirect Effects**									
5.CD -> MOT -> INTPERF	0.158	0.159	0.020	7.852	**0.000** **	0.177	0.102		

Source: Field survey (2021);

**p<0.000,

*p<0.05 supported.

### Path analysis and hypotheses testing for the model

**Fig 3:**
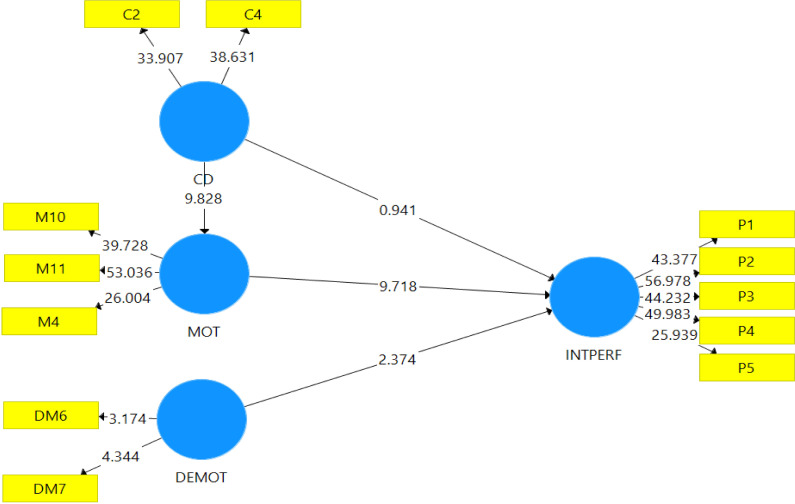
Bootstrapping results for path analysis. Source: Field survey (2021).

Before results for path analysis are presented, a bootstrapping sequence of 5000 samples utilised in the PLS according to the recommendation of [38] was carried out. The results, as presented in Fig 3, confirmed the significance of the hypothesised paths.

### Results for hypotheses testing

Table 4 presents the detailed results of path significance determined from the PLS bootstrapping sequence. The results revealed that the structural model explained about 0.137 variances in intention to participate in international assignments (INTPERF), and 0.154 variances in motivation (MOT) as expressed under the R^2^. The adjusted r-square in Table 4 further supports the results for the R2. The approximate 13.7 percent variance explained by the independent variables of the study suggests that there were other factors that influenced the intention to accept an international assignment among workers in developing economies that were not included in the model.

The results for the path analysis, as presented in Table 4, suggest that four out of the five hypotheses guiding the study were supported since they attained statistical significance. That is, cultural disposition (CD) had a statistically significant relationship with motivation to accept an international job or assignment (MOT) at (*β* = 0.393, t = 10.235, p = 0.000) for hypothesis two. There was a significant statistical relationship between demotivation for international assignment (DEMOT) and intention to participate in an international assignment (INTPERF) at (*β* = 0.106, t = 2.604, p = 0.009) for hypothesis three; and motivation for expatriation (MOT) and expatriate’s intention to participate in an international assignment (INTPERF) at (*β* = 0.137, t = 6.902, p = 0.000) for hypothesis four of the study. The last hypothesis was supported because it attained statistical significance. That is, motivation (MOT) statistically and significantly mediated the relationship between cultural disposition (CD) and expatriate intention to participate in an international assignment (INTPERF) for hypotheses five at (*β* = 0.158, t = 7.852, p = 0.000). Meanwhile, the study found a non-statistical significant relationship between cultural disposition (CD) and expatriate intention to participate in an international assignment (INTPERF) at (*β* = 0.015, t = 0.394, p = 0.693) for hypothesis one.

### Importance of performance map analysis (IPMA)

PLS Importance Performance Map Analysis (IPMA) was conducted in this study to further check the PLS estimates of the structural model variable relationships, and the results are presented in Table 5. The analysis compares the constructs’ total effect to that of its performance to determine the most relevant to consider for policy decisions. This was done based on [37] argument that the total effects represented the sum of direct and indirect effects; hence, the unstandardised effects were relied upon by the IPMA to enable a “ceteris paribus” interpretation of predecessor constructs’ impact on the target construct.

**Table 5:** Importance performance index values and total effects for INTPERF.

	Total Effect (*Importanc*e)	Index Values (Performance)
CD	0.173	74.117
DEMOT	0.105	31.921
MOT	0.399	68.700

Source: Field survey (2021).

**Fig 4:**
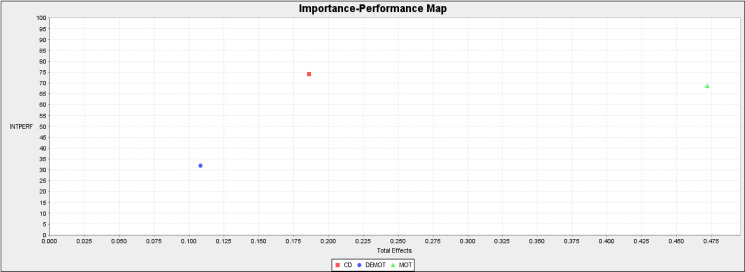
IPMA for INTPERF. Source: Field survey (2021).

**Fig 5:**
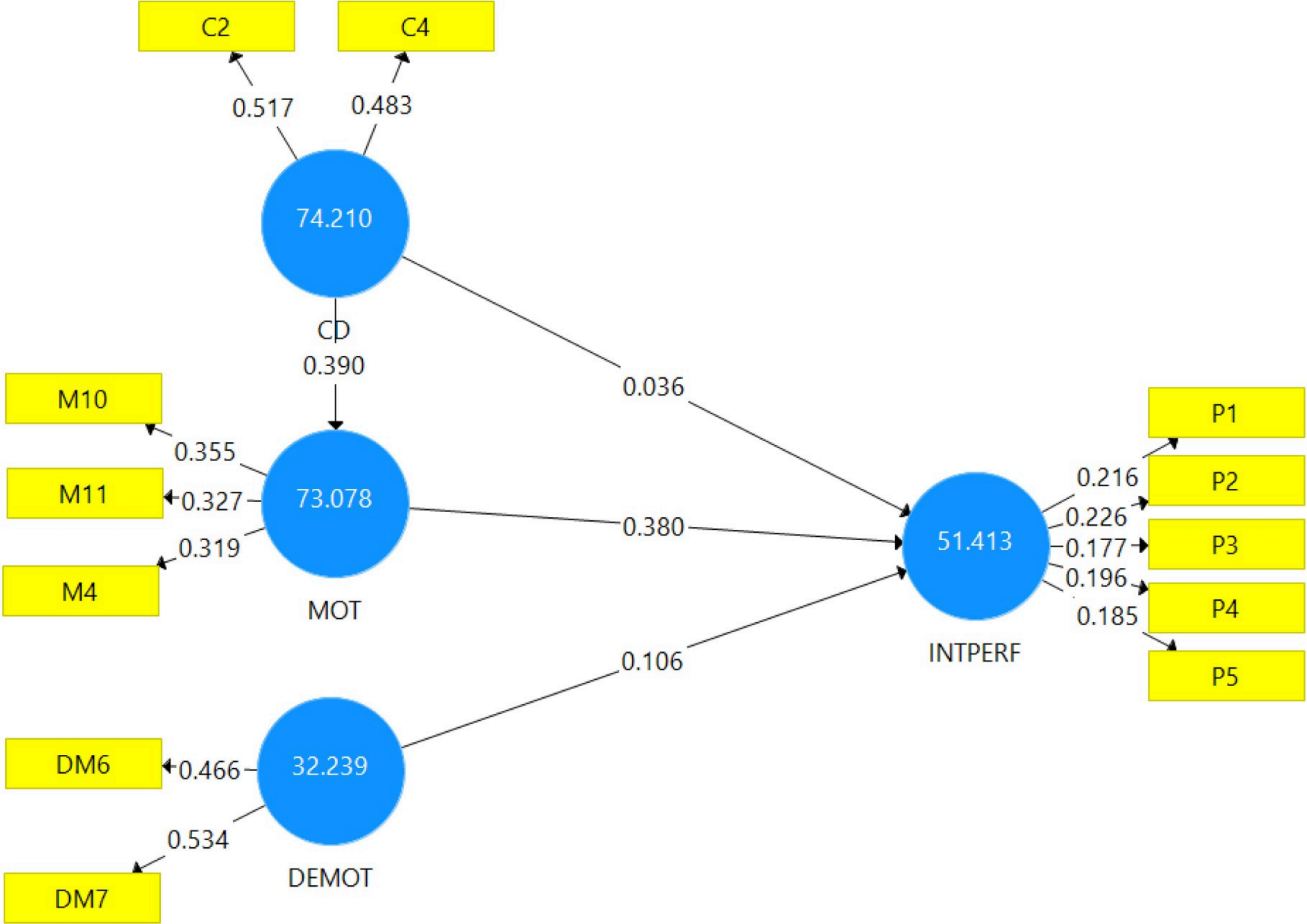
IPMA path analysis. Source: Field survey (2021).

This meant that the size of the total unstandardised effect increased the performance of the target construct when there was an increase in certain predecessor construct’s performance. The results, as presented in Table 5, show the IPMA values for expatriates intention to participate in an international assignment (INTPERF). From Table 5, the construct with the highest and strongest performance value was cultural disposition (CD), with a value of 74.117. However, cultural disposition was not the most relevant in predicting expatriate intention in the model since its importance value was the second-highest (0.173). Therefore, the model’s most relevant predictor of expatriate performance was rather a motivation to take on expatriate assignment (MOT) with the highest value of 0. 399. The results are further corroborated by the additional information provided in Fig 4. It is obvious from Fig 5 that MOT was the most relevant predictor of expatriate performance in international assignments, followed by CD.

### Graphical representation of the PLS IPMA path results

Fig 5 further presents the results for the graphical representation of the IPMA path analysis for the PLS path model. This presentation was based on [37] argument that there are differences between the graphical PLS-SEM results and the graphical representation of IPMA in PLS outputs. The values of performance of individual independent variables IPMA are shown instead of the dependent variables’ values of R^2^ in the PLS path model. Additionally, [37, 41] believed the IPMA results highlighted the unstandardised and recalled the outer weights of the measurement models (formative and reflective) and not the standardised outer loading or weights. Therefore, the beta values in the outer model reported in Fig 5 indicate the importance of individual items to the construct and not the loading. Thus, in this study, the results of the beta values highlighted in the outer model in Fig 5 revealed each item’s importance to the construct and not the loading. Additionally, inner values presented in Fig 5 further revealed the performance values of each of the variables of the study to the endogenous variable and not the total variance explained.

## Discussion of results

The discussion of the results session focuses on the five hypotheses guiding the study based on the three independent variables of the study (cultural disposition, motivators and demotivators) related to the dependent variable-intention to accept an international assignment. The findings for hypothesis one, that there was a non-statistical significant relationship between cultural disposition and expatriate intention to participate in an international assignment, can be explained further. The result suggests that an expatriate’s experience in working in different cultures in terms of weather, food, and clothing, and ability to work with people of different cultures were good for an expatriate assignment but do not solely influence their intention. The result suggests that cultural disposition alone is not enough to influence the intention of expatriates to accept an international assignment. Thus, from the individual workers’ and developing economy perspectives, a cultural disposition is not enough to ensure significant relations with an intention to accept expatriate assignment. The results thus disagree with that of [6], who found that cultural disposition is a strong predictor of intention in an international assignment.

It is important to note that the non-significant relationship established between cultural disposition and expatriate intention in hypothesis one is further and better explained by hypothesis five. The findings for hypothesis five suggest that motivation statistically and significantly mediates the relationship between cultural disposition and the expatriate’s intention to participate in international assignments. The results mean that expatriates need to be motivated to take up an international assignment in addition to their cultural disposition before they can accept an international assignment. Thus, an employee must be convinced or motivated that in addition to cultural disposition, an international assignment will help him/her to meet career goals, bring him/her recognition, opportunities, and social rewards, will empower him/her for future higher assignments, to experience higher performance on the international assignment. The results mean that motivation is a potent mediator between cultural disposition and acceptance of an international assignment from an individual and developing economy perspectives. The findings agree with the findings of [31] that expatriate motivation to take up an international assignment influences their performance.

Findings for hypothesis two, that there was a statistically significant relationship between cultural disposition and motivation for an international assignment, need further explanation. The results can be explained that expatriates will be motivated to take up an international assignment when they have experience in working in different cultures, are able to work with people from different cultures and when one has the ability to learn other languages. Thus, motivation for an international assignment is highly dependent on one’s cultural disposition. To increase workers’ motivation for international assignments in other parts of the world, employers will need to pay attention to exposing workers to different cultures. This could take the form of organising cross-cultural training for workers expected to take up international assignments. This finding of the study equally corroborates the findings of [29] that cultural disposition is a strong predictor of motivation to take up an international assignment.

The findings that demotivation among workers’ assignments significantly predicted the intention to take up an international assignment have several dimensions to be explained. The results suggest that there are factors that can cause disaffection toward opting for an international assignment. Thus, expatriates’ intentions and performance could suffer depending on the level of dissatisfaction. A low dissatisfaction could lead to a low effect on intention, and a high dissatisfaction could lead to a high effect on the intention of expatriates. Thus, expatriates’ intentions could be affected if the worker’s spouse cannot accompany them on an international assignment and cannot get the right person to take care of the family at home while on an international assignment. Other dissatisfiers that could influence acceptance for expatriate assignment and intention to accept an international assignment were the negative impact of an internal assignment on children’s education and not being able to readjust after the expiration of an international assignment. The findings of this study agree with the findings of [29, 39] that demotivation among workers affects acceptance of the expatriate assignment and the performance of expatriates.

The last finding of the study, that motivation for expatriate assignment significantly influences intention to participate in an international assignment, also need to be explained further. The results mean that workers will be motivated to take up an international assignment if motivators are attached to the appointment. Thus, if a worker is forced or coerced to take up an international assignment against their will, this could affect the intention and performance. The results further suggest that it is not enough to use an organisational perspective to determine intention for international assignments as captured in previous studies. Rather, individual workers’ motivation for international assignment is key for successful international assignments among expatriates. Thus, the findings of [26] that motivation among workers to take expatriate jobs greatly influence their intention and performance in an international assignment is upheld by the findings of this study. Though previous studies found a significant relationship between cultural disposition and intention to accept an international assignment. this study revealed and added to knowledge that cultural disposition is not adequate in influencing intention for expatriate assignments, but rather does so through motivation.

### Theoretical implications

The findings of this study have several theoretical implications for Hofstede’s Cultural Model. This study is premised on a national culture that is generally characterised by high power distance culture, collectivism culture, feminine culture, high uncertainty avoidance culture and short-term orientation culture, as captured in the five dimensions of the theory. These characteristics need to be considered and respected by human resource managers when recruiting and training people for an international assignment in Ghana or sending a Ghanaian on an expatriate mission. This is because the findings of this study have confirmed certain hidden characteristics in theory. These are cultural matters in international business. There is a statistical difference between the populations of two countries or ethnic groups. The dimensions reflect stable national differences, implying that cultures tend to move together in the same cultural direction. Thus, cultural adaptation and integration become easier for an expatriate assignment in similar cultures.

### Practical implications

The findings of this study have practical implications for human resource managers of multinational organisations. The human resource of every organisation remains one of the most important factors for international organisations. The first implication of this study relates to the relevance of cultural disposition for intention and expatriate performance. It suggests that human resource managers need to expose their workers to other cultures to prepare individuals to accept and work in other cross cultures. Cross-cultural exposures can come in the form of job rotations, transfers and teamwork. These can help to expose the individual to local cross-cultural elements, which could serve as a launchpad for accepting to work on an international assignment.

Another implication of the results of this study also relates to the motivation level of workers to accept an international assignment. It calls for ensuring that better packages are associated with international appointment. This could relate to remuneration, jobs for spouses, provision for children’s education, intense cross-cultural training and allowing workers to have international exposure by visiting the country of the international assignment before finally taking the appointment. Closely related to the motivation was also the demotivation for international appointments. Failure on the part of human resource managers of international firms to provide adequate information on the international appointment and exposure of workers to other cultures can result in two things. That is, it can lead to an unwillingness to accept an international assignment and greater chances of expatriate failure or poor performance. The policy implication of the findings of this study was that culture, motivation and demotivation should be taken very seriously by all multinational organisations for greater acceptance and for an international assignment.

## Conclusion and recommendations

This study examined the motivation and demotivation for accepting expatriate assignments among workers in Ghana. Previous studies have examined these factors from organisational perspectives. However, the present study examined these factors from individual workers and from developing economy perspectives. Therefore, it can be concluded from the individual and developing economy perspectives, based on the findings of this study, that cultural disposition influences motivation for accepting an international assignment. Motivation and demotivation among workers were also found to have had a statistically significant relationship between expatriate intention and significantly mediated the relationship between cultural disposition and expatriate intention to participate in an international assignment. However, cultural disposition was found to have a non-significance relationship with expatriates’ intention to accept an international assignment. The contribution of this study to knowledge is from individual and developing economy perspectives. That is, cultural disposition alone is not potent to relate very well to acceptance of international assignment among individual workers in a developing economy like Ghana. Rather, cultural disposition becomes a significant factor when associated with motivators.

These conclusions call for a recommendation for managers of multinational organisations and human resource managers. Therefore, it is suggested that human resource managers expose workers to cross-cultural training through job rotations, working in teams, and experiential training. It is expected that such opportunities will reduce culture shock and prepare individuals for an international assignment. It is also recommended that international assignments should be made very attractive to be able to appeal to workers to be willing to accept such job openings. Expatriates should get more recognition, opportunities, social rewards, professional skills, attractive remuneration and other benefits, among others, attached to the expatriate job. The expatriate should also be prepared well for repatriation after the end of an international assignment.

Additionally, human resource managers should deal with demotivators with an international assignment. These demotivators relate to alienation from family, not getting the right person to take care of the family while on the international assignment, and differences in religious beliefs between host and home countries. This can be addressed by ensuring that expatriates are sent to their country or continent of their choice or similar exposure. Allowing a spouse to accompany the expatriate and providing jobs for spouses, as well as paying academic bills for children’s education for the expatriate in the foreign country, will be very helpful in addressing some of the challenges that relate to demotivation.

### Limitations and suggestions for further studies

This study was limited to a quantitative approach and failed to capture the qualitative dimensions. The focus of this study was on the general motivation and demotivation among workers in Ghana toward an international assignment. It did not focus on only workers already on an international assignment. Future studies can pursue these dimensions. The analysis of this study did not consider the demographic characteristics of respondents in terms of gender and sector of employment. Other further studies can consider these perspectives. A comparative study between two or more countries will also be very revealing.

## Supporting information

S1 Appendix Descriptive results of the study. (DOCX)

S2 Appendix Outer VIF values. (DOCX)

S1 Data (CSV)

## List of abbreviations

CFA: Confirmatory Factor Analysis
CD: Cultural Disposition
HTMT: Heterotrait-Monotrait Ratio
MOT: Motivation to accept an international assignment
DEMOT: Motivation to accept an international assignment Dem
INTPERF: Intention for t an international assignment
PLS: Partial Least Squares
PLS-SEM: Partial Least Squares Structural Equation Modelling
SEM: Structural Equation Modelling
VIF: Variance Inflated Factor
