# Supplementary material for: Intention for international assignment among workers in Ghana: Modelling the role of motivators, demotivators and cultural disposition
Source: PLoS One. 2023 May 4;18(5):e0284615. doi: 10.1371/journal.pone.0284615 (PMC10159112; doi:10.1371/journal.pone.0284615)
Supplement: S1 Appendix — (DOCX) [file pone.0284615.s001.docx]

**Appendix A: Descriptive results for independent variables of the study**

| **Cultural disposition** | **N** | **Minimum** | **Max.** | **Mean** | **Std. Dev.** |
| --- | --- | --- | --- | --- | --- |
| I can work with people of different cultures | 723 | 1.00 | 4.00 | 3.3264 | .74893 |
| I can adapt to different cultures with ease in terms of weather, food, and clothing, among others | 723 | 1.00 | 4.00 | 3.1189 | .84847 |
| I have experience living and working in different cultures | 723 | 1.00 | 4.00 | 2.8174 | 1.01232 |
| I am currently working in different cultures other than my native culture | 723 | 1.00 | 4.00 | 2.7261 | 1.02852 |
| I can learn other languages with ease | 723 | 1.00 | 4.00 | 2.6874 | .88019 |

**Motivation**

| **Motivation to accept intentional assignment** | **N** | **Min.** | **Max.** | **Mean** | **Std. Dev.** |
| --- | --- | --- | --- | --- | --- |
| Because an international assignment will empower me for future higher as-signments | 723 | 1.00 | 4.00 | 3.2642 | .86366 |
| To learn more professional skills while on an international assignment | 723 | 1.00 | 4.00 | 3.2393 | .80477 |
| Because I find the experience of how to live in and work with different cul-tures valuable | 723 | 1.00 | 4.00 | 3.1189 | .81347 |
| Because the international assignment will help me meet my career goals | 723 | 1.00 | 4.00 | 3.0664 | .86548 |
| Because the financial and other benefits are attractive | 723 | 1.00 | 4.00 | 2.9156 | .86631 |
| Because I get more recognition, opportunities, and social rewards when I live and work internationally | 723 | 1.00 | 4.00 | 2.8755 | .91069 |
| Because I like being on an international assignment an adventure | 723 | 1.00 | 4.00 | 2.7773 | .87845 |
| Because I may end up regretting not going if I turned it down | 723 | 1.00 | 4.00 | 2.4509 | .95538 |
| Because the organization expects its workers to accept international assignments | 723 | 1.00 | 4.00 | 2.4066 | .93445 |
| Because I want to have the respect of family and friends as an international assignee | 723 | 1.00 | 4.00 | 2.2628 | .94888 |
| To avoid feeling bad since my spouse wanted to go | 723 | 1.00 | 4.00 | 2.0775 | .92193 |

**Demotivation**

| Demotivation to accept an international assignment | N | Minimum | Maximum | Mean | Std. Dev. |
| --- | --- | --- | --- | --- | --- |
| My children’s education and upbringing will suffer negatively in my absence | 723 | 1.00 | 4.00 | 2.3804 | .99194 |
| Because I will not get the right person to take care of my family at home while I am on an international assignment | 723 | 1.00 | 4.00 | 2.2241 | .93241 |
| Because I will be disconnected from my friends and family back home | 723 | 1.00 | 4.00 | 2.1328 | .90799 |
| Because of unfavorable weather conditions in the host/foreign country for an international assignment | 723 | 1.00 | 4.00 | 2.0858 | .92419 |
| Because my spouse cannot accompany me on an international assignment | 723 | 1.00 | 4.00 | 2.0401 | .93844 |
| Because of differences in religious beliefs between host and home countries | 723 | 1.00 | 4.00 | 2.0373 | .89163 |
| Because of an inferior educational system in the foreign country for the international assignment | 723 | 1.00 | 4.00 | 1.8866 | .87017 |
| Because I will not be able to readjust upon return to the home country after the expiration of an international assignment | 723 | 1.00 | 4.00 | 1.8755 | .85742 |
| Because I am a woman and my culture does not support staying away from the man | 723 | 1.00 | 4.00 | 1.7469 | .91060 |

Source: Field survey (2021)
