# Supplementary material for: Intention for international assignment among workers in Ghana: Modelling the role of motivators, demotivators and cultural disposition
Source: PLoS One. 2023 May 4;18(5):e0284615. doi: 10.1371/journal.pone.0284615 (PMC10159112; doi:10.1371/journal.pone.0284615)
Supplement: S2 Appendix — (DOCX) [file pone.0284615.s002.docx]

**Appendix B: Outer VIF Values**

| Items | VIF |
| --- | --- |
| C2 | 1.298 |
| C4 | 1.298 |
| DM6 | 1.216 |
| DM7 | 1.216 |
| M10 | 1.615 |
| M11 | 1.705 |
| M4 | 1.275 |
| P1 | 2.100 |
| P2 | 2.259 |
| P3 | 2.246 |
| P4 | 2.220 |
| P5 | 1.589 |

Source: Field survey (2021)
